# Regulation of photosensation by hydrogen peroxide and antioxidants in *C*. *elegans*

**DOI:** 10.1371/journal.pgen.1009257

**Published:** 2020-12-10

**Authors:** Wenyuan Zhang, Feiteng He, Elizabeth A. Ronan, Hongkang Liu, Jianke Gong, Jianfeng Liu, X.Z. Shawn Xu

**Affiliations:** 1 College of Life Science and Technology, Key Laboratory of Molecular Biophysics of MOE, Huazhong University of Science and Technology, Wuhan, Hubei, China; 2 Life Sciences Institute, University of Michigan, Ann Arbor, Michigan, United States of America; 3 Department of Molecular and Integrative Physiology, University of Michigan Medical School, Ann Arbor, Michigan, United States of America; Brown University, UNITED STATES

## Abstract

The eyeless *C*. *elegans* exhibits robust phototaxis behavior in response to short-wavelength light, particularly UV light. *C*. *elegans* senses light through LITE-1, a unique photoreceptor protein that belongs to the invertebrate taste receptor family. However, it remains unclear how LITE-1 is regulated. Here, we performed a forward genetic screen for genes that when mutated suppress LITE-1 function. One group of *lite-1* suppressors are the genes required for producing the two primary antioxidants thioredoxin and glutathione, suggesting that oxidization of LITE-1 inhibits its function. Indeed, the oxidant hydrogen peroxide (H_2_O_2_) suppresses phototaxis behavior and inhibits the photoresponse in photoreceptor neurons, whereas other sensory behaviors are relatively less vulnerable to H_2_O_2_. Conversely, antioxidants can rescue the phenotype of *lite-1* suppressor mutants and promote the photoresponse. As UV light illumination generates H_2_O_2_, we propose that upon light activation of LITE-1, light-produced H_2_O_2_ then deactivates LITE-1 to terminate the photoresponse, while antioxidants may promote LITE-1’s recovery from its inactive state. Our studies provide a potential mechanism by which H_2_O_2_ and antioxidants act synergistically to regulate photosensation in *C*. *elegans*.

## Author summary

The nematode *C*. *elegans* possesses a unique photoreceptor protein, LITE-1, which mediates a light-avoidance behavior upon light exposure. *C*. *elegans* avoids short-wavelength light, particularly UV light, providing a potential mechanism by which worms escape from the dangerous UV rays in the sunlight. However, it is not clear how LITE-1 is regulated. Here, we performed a genetic screen to identify genes regulating LITE-1. We uncovered six genes that when mutated suppress LITE-1 function. All these genes are involved in regenerating cellular antioxidants that function to clear reactive oxygen species, particularly hydrogen peroxide (H_2_O_2_), suggesting that the function of LITE-1 is vulnerable to H_2_O_2_. Indeed, we show that H_2_O_2_ exposure inhibits LITE-1 function, while antioxidants promote it. Notably, other sensory functions are relatively less sensitive to H_2_O_2_. As UV light illumination is known to generate H_2_O_2_ within the cell, this provides a potential mechanism to turn off LITE-1. Our results uncover a potential mechanism of LITE-1 regulation, where antioxidants and oxidants act to promote and suppress LITE-1 function, respectively.

## Introduction

Photosensation is a universal phenomenon found in nearly every organism, including bacteria and plants [1,2]. *C*. *elegans*, though lacking a specialized light-sensing organ, engages in robust avoidance behavior in response to short-wavelength light, particularly UV light, providing a potential mechanism for worms to avoid lethal doses of UV light in the sunlight [3–7]. Such phototaxis behavior is mediated by a group of photoreceptor neurons [3], and the underlying molecular light sensor is LITE-1 [5].

LITE-1 is a member of the invertebrate gustatory receptor (GR) family [5]. Members in this family do not all function as chemoreceptors. For example, in addition to LITE-1 that senses light, some GR members function as thermoreceptors [8]. LITE-1 possesses several unique features distinct from other types of photoreceptors found in the animal kingdom such as opsins and cryptochromes. For example, LITE-1 is predicted to possess seven-transmembrane (7-TM) domains but with an inversed membrane topology [5]. LITE-1 is highly efficient in capturing photons, with an efficiency 10–100 times that of opsins and crytochromes [5]. LITE-1 strictly relies on its conformation for photoabsorption and may not require a prosthetic chromophore [5]. Despite these interesting features, it remains unclear how LITE-1 functions.

To identify genes regulating LITE-1, here we performed a genetic screen for mutants that suppress LITE-1 function. Among *lite-1* suppressors, one major class of genes encode proteins that function to regenerate thioredoxin and glutathione, the two primary antioxidants in the cell. This suggests that protection of LITE-1 from oxidization by antioxidants is crucial for the function of LITE-1. In support of this model, we found that treating wild-type worms with hydrogen peroxide (H_2_O_2_), an oxidizing agent, inhibits LITE-1 function and suppresses phototaxis behavior, while antioxidant treatment of *lite-1* suppressor mutants can rescue the mutant phenotype. We also found that *lite-1* suppressor mutants are defective in recovering from light-induced loss of photosensitivity, suggesting that antioxidants facilitate the recovery of LITE-1 from its inactive state. Further functional analysis in photoreceptor neurons indicate that H_2_O_2_ and antioxidant inhibits and promotes LITE-1-mediated photocurrent, respectively. Interestingly, other sensory functions are less vulnerable to H_2_O_2_ than photosensation, revealing a relatively higher sensitivity of photosensation to H_2_O_2_ inhibition. Given that UV light illumination produces H_2_O_2_, our data suggest a model that upon light activation of LITE-1, H_2_O_2_ produced by light then inactivates LITE-1 to terminate the photoresponse, while antioxidants may facilitate the recovery of LITE-1 from its inactive state.

## Results

### A genetic screen for *lite-1* suppressors

To identify genes regulating LITE-1, we sought to design a genetic screen for mutant worms that fail to respond to light. Our previous effort of screening for phototaxis mutants, however, only isolated *lite-1* [4]. This might be caused by the fact that multiple types of photoreceptor neurons can drive phototaxis behavior [3,4]. In addition, mutants defective in regulating LITE-1 function may not exhibit a phototaxis behavioral phenotype as severe as *lite-1* itself. As such, a more sensitive and/or quantitative assay is needed to identify genes regulating LITE-1.

To design such a screen, we took advantage of the fact that LITE-1 can be functionally expressed ectopically. For example, expression of *lite-1* as a transgene in light insensitive neurons and body-wall muscles confers light sensitivity to these otherwise light insensitive cells/tissue [4–6]. In particular, we showed that LITE-1 protein purified from LITE-1-expressing muscles is highly efficient in absorbing photons [5]. In addition, light illumination induces contraction of muscles ectopically expressing LITE-1, leading to paralysis [4–6]. We thus decided to use light-induced contraction of LITE-1-expressing muscles (i.e. paralysis) as a readout to screen for suppressor mutants.

**Fig 1:**
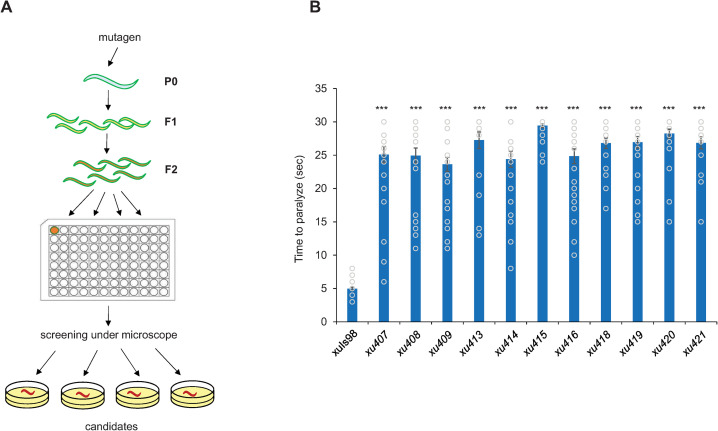
A forward genetic screen identifies *lite-1* suppressor mutants. (A) Schematic describing the design of the screen. Worms ectopically expressing *lite-1* in body-wall muscles were mutagenized with EMS and their progeny was screened for resistance to UV light-induced paralysis. Progeny was screened in a liquid assay with 96-well plates. (B) *lite-1* suppressor screening identified 17 mutants that are severely defective in light-induced paralysis. Bar graph shows mean time to paralyze upon exposure to light for the 11 mutants whose mutations were mapped in subsequent analysis. All strains carry the transgene *xuIs98* that ectopically expresses *lite-1* in body-wall muscles. Error bars: SEM. n≥20. ***p<0.0005 (ANOVA with Bonferroni test).

**Fig 2:**
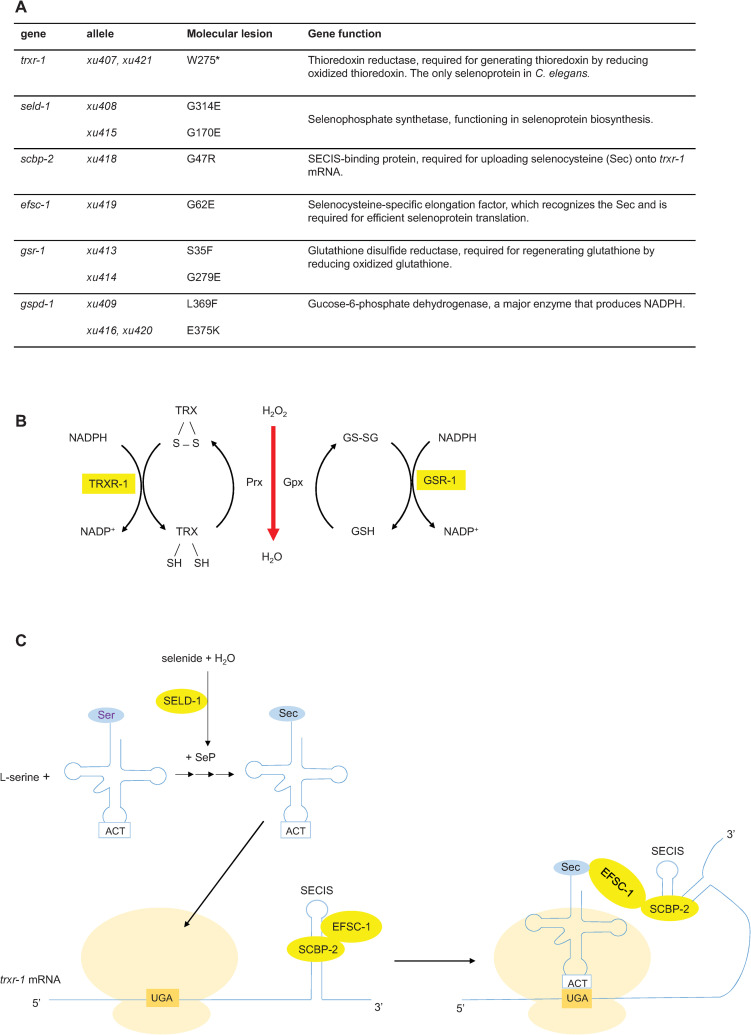
*lite-1* suppressors include genes involved in regenerating the antioxidants thioredoxin (TRX) and glutathione (GSH). (A) Whole-genome sequencing mapped the mutation in 11 suppressor strains to six different genes, all of which are involved in regenerating the primary cellular antioxidants thioredoxin (TRX) and glutathione (GSH). Four genes have multiple mutant alleles. Two mutants bear the same molecular lesion in each of *trxr-1* and *gspd-1*, though they came from different rounds of screens. (B) Suppressors isolated include genes required for clearing H_2_O_2_ via antioxidants. Both peroxiredoxin (Prx) and glutathione peroxidase (Gpx) function to directly convert H_2_O_2_ to H_2_O. Prx and Gpx regeneration requires reduction via thiol-disulfide exchange, with Prx regeneration primarily requiring TRX. Gpx regeneration requires GSH. TRXR-1 and GSR-1 are required for regenerating TRX and GSH, respectively. NADPH functions as a cofactor (electron donor) to regenerate both TRX and GSH. Highlighting denotes genes isolated in the *lite-1* suppressor screen. (C) *lite-1* suppressors include genes required for TRXR-1 protein synthesis. As the sole enzyme that regenerates TRX in the cytosol, mutations that result in the loss of TRXR-1 also suppress *lite-1*. As the only selenoprotein in *C*. *elegans*, TRXR-1 protein synthesis requires the synthesis and in cooperation of selenocysteine-tRNA (Sec-tRNA^Sec^) during translation. Production of the selenophosphate is dependent on SELD-1, which is required for exchanging the serine residue on L-serine for selenocysteine (Sec). Once synthesized, Sec-tRNA^Sec^ recognizes the UGA stop codon in *trxr-1* mRNA. The Sec insertion sequence (SECIS) element, a cis-acting stem-loop structure present in selenoprotein mRNAs, is required for the insertion of Sec during translation [12]. This insertion requires SECIS to bind SCBP-2, which recruits EFSC-1 which can in turn bind the Sec-tRNA^Sec^ and aids in the incorporation of Sec during translation [12]. Highlighting denotes genes isolated in the *lite-1* suppressor screen.

We screened for mutants that showed resistance to light-induced paralysis in a liquid assay using 96-well plates (Fig 1A). After screening ~25,000 F2 worms (from ~4000 F1), we isolated 17 *lite-1* suppressors. By whole-genome sequencing, we mapped the mutation in 11 out of 17 suppressors to six different genes (Figs 1B and 2A). Four genes have multiple mutant alleles, and some mutants bearing the same molecular lesion were isolated twice, though they came from different rounds of screens (Fig 2A).

### *lite-1* suppressors include genes involved in regenerating the antioxidants thioredoxin and glutathione

Among the six genes identified from our screen, they share one common feature: they all are involved in regenerating thioredoxin (TRX) and glutathione (GSH), the two primary antioxidants produced by the cell (Fig 2A and 2B). These two antioxidants play a key role in clearing reactive oxygen species (ROS), particularly hydrogen peroxide (H_2_O_2_) in the cell (Fig 2B) [9]. Thioredoxin also functions as a reductase to facilitate the reduction of other oxidized proteins [10,11]. We classify the six genes into three sub-groups as follows:

### Thioredoxin (TRX) pathway

*trxr-1*, *seld-1*, *scbp-2*, and *efsc-1*. *trxr*-*1* encodes the worm homolog of thioredoxin reductase, the sole selenoprotein in the worm genome [9,12]. This enzyme is required for regenerating thioredoxin by reducing oxidized thioredoxin (Fig 2B) [10,13]. As a selenoprotein, TRXR-1 protein translation requires the synthesis and incooperation of selenocysteine-tRNA (Sec-tRNA^Sec^) that is recognized by the UGA stop codon in *trxr-1* m RNA (Fig 2C) [12,14]. Interestingly, the other three genes *seld-1*, *scbp-2* and *efsc-1* all encode key components in this process (Fig 2C) [12,14].

### Glutathione (GSH) pathway

*gsr-1*. *gsr-1* encodes the worm homolog of glutathione reductase (Fig 2A and 2B). This enzyme is required for regenerating glutathione by reducing oxidized glutathione (Fig 2B) [9].

### NADPH production

*gspd-1*. To regenerate thioredoxin and glutathione, both TRXR-1 and GSR-1 require NADPH as a cofactor (electron donor) (Fig 2B) [9]. *gspd-1* encodes the worm homolog of glucose-6-phosphate dehydrogenase that catalyzes the production of NADPH (Fig 2A). This is the major source of NADPH in the cytosol [15].

**Fig 3:**
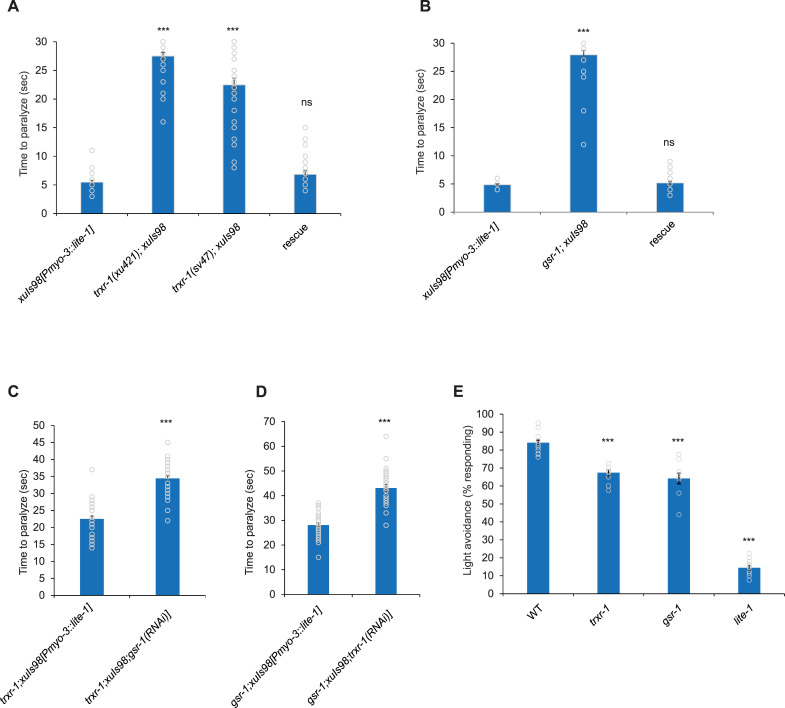
*trxr-1* and *gsr-1* mutant worms are defective in photosensation. (A) TRXR-1 mediates the LITE-1-dependent, light-evoked paralysis phenotype. *trxr-1(xu421)* and *trxr-1(sv47)* mutants were defective in the response. Transgenic expression of wild-type *trxr-1* cDNA in body-wall muscle using the *myo-3* promoter rescued the mutant phenotype of *trxr-1(sv47)* worms. Error Bars: SEM. n = 30. ***p<0.0005 (ANOVA with Bonferroni test). (B) GSR-1 mediates the LITE-1-dependent, light-evoked paralysis phenotype. *gsr-1(xu414)* mutant worms were severely defective in the response. Transgenic expression of wild-type *gsr-1* cDNA in body-wall muscles using the *myo-3* promoter rescued the mutant phenotype. Error Bars: SEM. n = 30. ***p<0.0005 (ANOVA with Bonferroni test). (C) *gsr-1(RNAi)*; *trxr-1(sv47)* worms are more defective than *trxr-1* single mutant in light-evoked, LITE-1 dependent paralysis. *gsr-1* RNAi was expressed as a transgene in body-wall muscles under the *myo-3* promoter. Error Bars: SEM. n≥30. ***p<0.0005 (t-test). (D) *trxr-1(RNAi); gsr-1(xu414)* worms are more defective than *gsr-1* single mutant in light-evoked, LITE-1 dependent paralysis. Error Bars: SEM. n≥30. ***p<0.0005 (t-test). (E) *trxr-1* and *gsr-1* mutant worms are defective in phototaxis behavior. A rapid light pulse elicits a robust reversal response in WT worms, and both *trxr-1(sv47)* and *gsr-1(xu414)* mutant worms were defective in this response. This defect is more severe in *lite-1(xu492)* worms, a deletion mutant of *lite-1*. Error Bars: SEM. n≥10. ***p<0.0005 (ANOVA with Bonferroni test).

In summary, all the six genes identified from our screen are involved in regenerating the antioxidants thioredoxin and glutathione. Among these six genes, *trxr-1* and *gsr-1* are a bit special in that they directly execute the regeneration of thioredoxin and glutathione, respectively (Fig 2B). Transgenic expression of wild-type *trxr-1* and *gsr-1* genes in body-wall muscles of *trxr-1* and *gsr-1* mutant worms rescued the light-evoked paralysis phenotype, indicating that they are the genes causing the phenotype (Fig 3A and 3B). We were unable to generate *trxr-1; gsr-1* double mutant, which might be caused by synthetic lethality of the two mutations. We thus took an alternative approach by knocking down *trxr-1* expression in body-wall muscles in *gsr-1* mutant background, and *vice versa*. *trxr-1(RNAi); gsr-1* and *trxr-1; gsr-1(RNAi)* worms both displayed a more severe phenotype than *trxr-1* and *gsr-1* single mutants (Fig 3C and 3D), indicating a functional redundancy of *trxr-1* and *gsr-1* in regulating LITE-1. By contrast, *trxr-1; prdx-2* double mutant worms showed a similar phenotype to *trxr-1* single mutant (S1 Fig), consistent with the notion that TRXR-1 and the peroxiredoxin act in the same pathway (Fig 2B). Due to the direct role of *trxr-1* and *gsr-1* in thioredoxin and glutathione regeneration, we decided to focus on characterizing these two genes.

### *trxr-1* and *gsr-1* mutant worms are defective in phototaxis behavior

As *trxr-1* and *gsr-1* form the primary pathways to regenerate antioxidants, their loss is expected to alter the redox state of the cell, making proteins in the cell more prone to oxidation by ROS such as H_2_O_2_. Interestingly, it has been reported that H_2_O_2_ can inactivate LITE-1 *in vitro* [5]. Notably, UV light illumination is known to generate H_2_O_2_ [7], which in turn may inactivate LITE-1. Thus, the observation that mutations in *trxr-1* and *gsr-1* suppressed light-induced paralysis mediated by *lite-1* muscle transgene is consistent with a model in which antioxidants produced by these two genes regulate LITE-1, probably by protecting it from inhibition by H_2_O_2_ *in vivo*.

**Fig 4:**
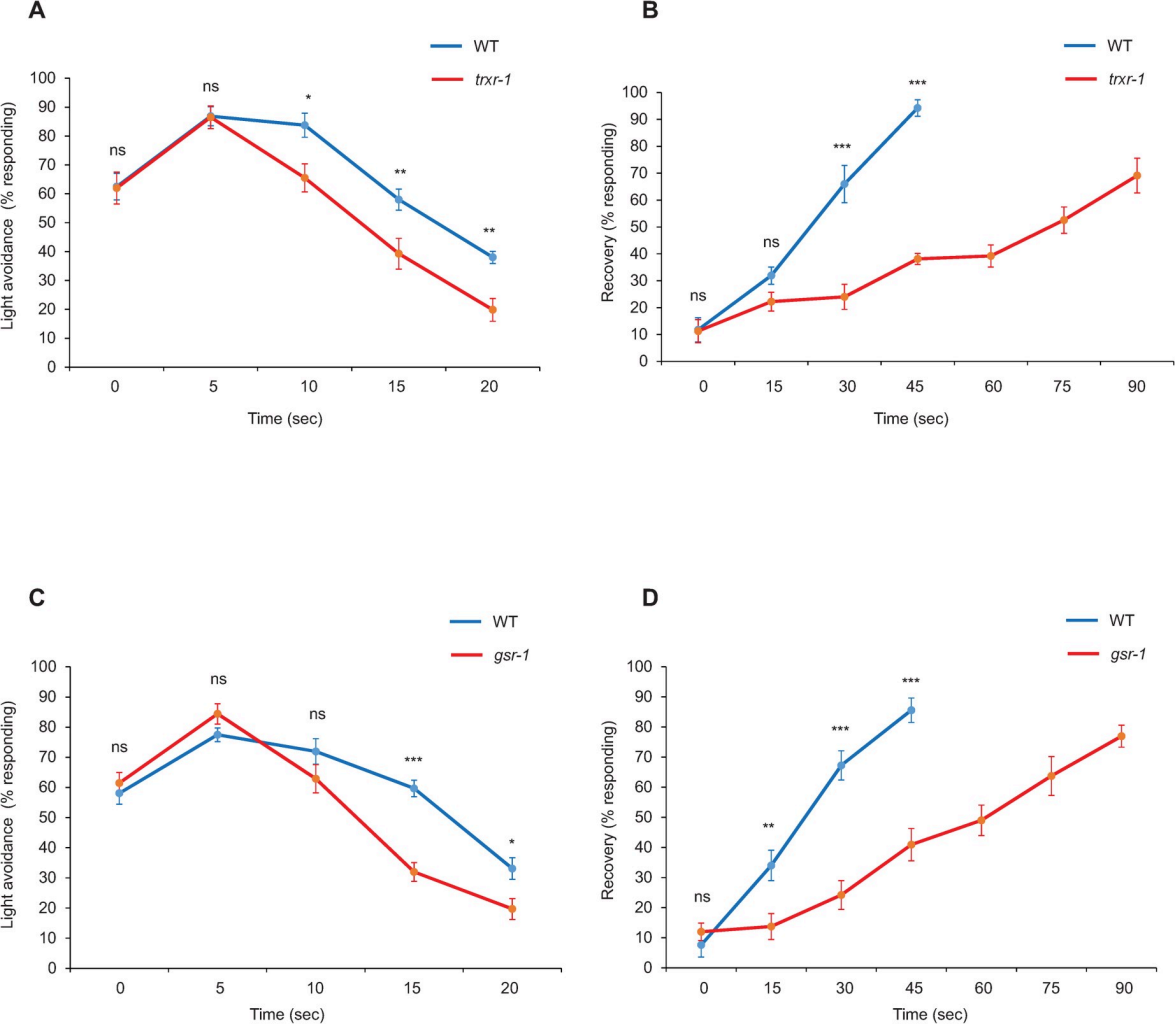
*trxr-1* and *gsr-1* mutant worms are defective in phototaxis response. (A and C) Pre-exposing worms to a light pulse of increasing durations prior to the phototaxis assay leads to a progressive decrease in phototaxis response. The x-axis denotes the duration of the light pulse to which the worm was pre-exposed. Following such pre-exposure, the worm was then tested for phototaxis behavioral response with a standard testing light pulse (2 sec). *trxr-1(sv47)* and *gsr-1(xu414)* mutants exhibited a faster decrease in phototaxis response compared to WT. Error Bars: SEM. n = 10. (A) **p<0.005, *p<0.05 (t-test). (C) ***p<0.0005, *p<0.05 (t-test). (B and D) Recovery from light-induced loss of photosensitivity is impaired in *trxr-1(sv47)* and *gsr-1(xu414)* mutants. Worms were pre-exposed to a 25 second UV light pulse, which led to a loss of photosensitivity. Such photo-insensitivity recovered over time. The x-axis denotes the duration of the time for which the worm was allowed to recover. After recovery, the worm was tested for phototaxis response with a standard testing light pulse (2 sec). Both mutations resulted in a slower pace of recovery compared to WT. Error Bars: SEM. n = 10. (B) ***p<0.0005 (t-test). (D) ***p<0.0005, **p<0.005 (t-test).

To test this, we first asked whether *trxr-1* and *gsr-1* are important for normal phototaxis behavior, as the light-induced paralysis assay does not measure LITE-1 function in a native setting. Indeed, both *trxr-1* and *gsr-1* mutant worms displayed a defect in phototaxis response (Fig 3E). The observed defect was not nearly as severe as *lite-1* mutant worms (Fig 3E). This might result from the functional redundancy of the two genes as suggested by our RNAi results (Fig 3C and 3D). We thus sought to design additional behavioral assays to test the role of *trxr-1* and *gsr-1* in photosensation. In our standard phototaxis assay, a 2 sec of light pulse was used to stimulate the worm [3]. If H_2_O_2_ produced by light illumination contributes to the inhibition of LITE-1, then a longer duration of light illumination would produce a higher amount of H_2_O_2_, which would presumably lead to more potent inhibition of the phototaxis response. To test this, we first pre-exposed worms to a light pulse of varying durations, and then scored the worm’s phototaxis behavior in response to a testing light pulse. As predicted, pre-exposing worms to a light pulse of increasing durations led to a progressive decrease in phototaxis response in wild-type worms (Fig 4A). We detected a faster decrease in phototaxis response in *trxr-1* and *gsr-1* mutant worms that were pre-exposed to light pulses, revealing a defect in *trxr-1* and *gsr-1* mutant worms (Fig 4A and 4C). To obtain additional evidence, we assessed how fast worms recovered from this light-induced inhibition of phototaxis behavior. We reasoned that in a more oxidized environment such as *trxr-1* and *gsr-1* mutant background, it shall take a longer time for worms to recover from light-induced loss of photosensitivity. Indeed, we found that *trxr-1* and *gsr-1* mutant worms exhibited a much slower recovery from light-induced photo-insensitivity compared to wild-type animals (Fig 4B and 4D), suggesting that antioxidants may facilitate the recovery of LITE-1 from its inactive state. These results together reveal a strong photosensation defect in *trxr-1* and *gsr-1* mutant worms.

Considering that the reduced level of antioxidants in *trxr-1* and *gsr-1* mutant worms may predispose all neurons/cells to oxidative damage, we wondered if these mutants display a general defect not restricted to phototaxis. To test this, we sampled several other sensory behaviors such as avoidance behavior (octanol), olfaction (IAA), and taste (NaCl), and observed no significant defect in *trxr-1* and *gsr-1* mutants (S2A, S2D, and S2G Fig). While this result by no means indicates a lack of other behavioral defects in *trxr-1* and *gsr-1* mutants, it suggests that the phototaxis defect observed in these mutants is not simply a non-specific phenomenon.

### Treating worms with H_2_O_2_ inhibits phototaxis behavior

**Fig 5:**
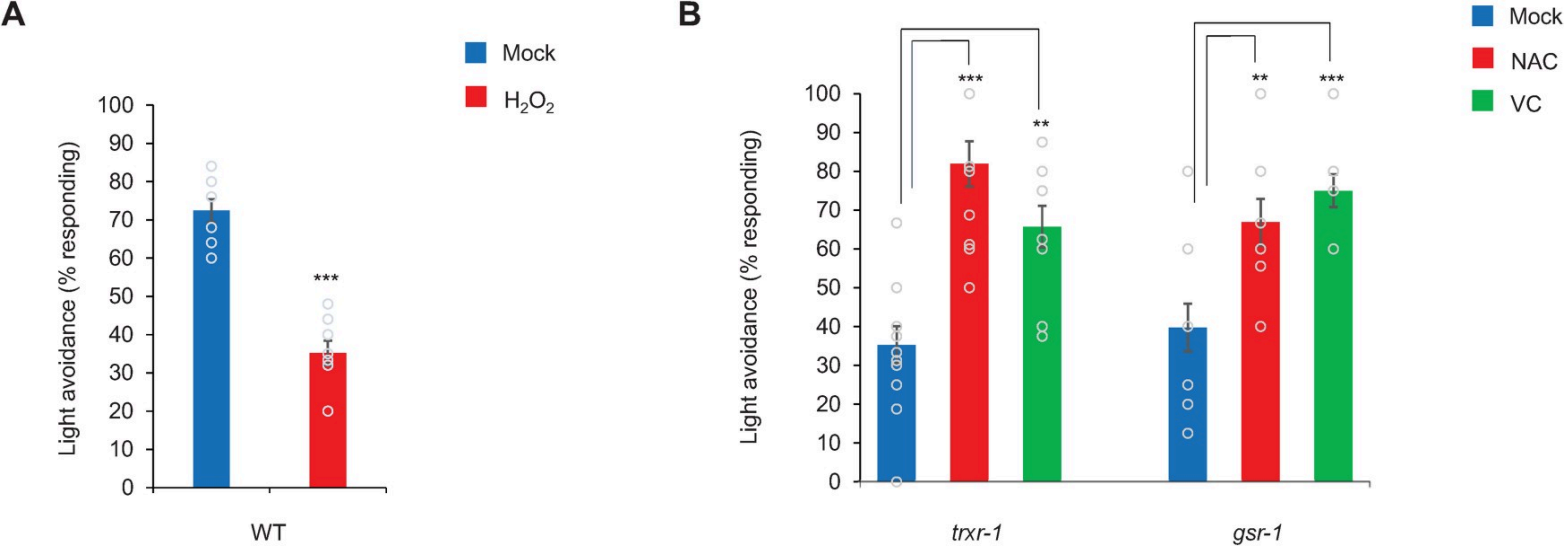
H_2_O_2_ inhibits phototaxis behavior, while antioxidants can rescue the phototaxis defect in *trxr-1* and *gsr-1* mutants. (A) H_2_O_2_ pre-treatment (500 μM) decreases the wild-type phototaxis response. Error Bars: SEM. n = 10. ***p<0.0005 (t-test). (B) Pre-treatment with antioxidants Vitamin C (VC, 10 mM) or N-acetyl Cysteine (NAC, 10 mM) potentiates the phototaxis response in *trxr-1(sv47)* and *gsr-1(xu414)* mutants. A 15 second pre-exposure light pulse was performed prior to the phototaxis assay. n = 10. ***p<0.0005 (ANOVA with Bonferroni test),**p<0.005 (ANOVA with Bonferroni test).

If H_2_O_2_ produced by light illumination inhibits phototaxis behavior, treating worms with H_2_O_2_ should exert an inhibitory effect on this behavior. We found that H_2_O_2_ treatment led to a marked decrease in phototaxis response in wild-type worms (Fig 5A). Paraquat treatment also decreased phototaxis response but to a lesser extent (S3 Fig). These results are consistent with the model that H_2_O_2_ inhibits LITE-1 function.

As a control, we examined whether H_2_O_2_ treatment compromises other avoidance behaviors. We found that the same H_2_O_2_ treatment (500 μM) had no notable effect on octanol avoidance response; nor did it affect osmotic avoidance behavior (S4A and S4B Fig). However, treating worms with higher concentrations of H_2_O_2_ (5 mM) impaired both octanol and osmotic avoidance behaviors (S4A and S4B Fig). While these results support a general detrimental role of H_2_O_2_ in impairing behavior, it points to an interesting observation that phototaxis behavior appears to be relatively more sensitive to H_2_O_2_.

### Antioxidant treatment can rescue the phototaxis defect in *trxr-1* and *gsr-1* mutant worms

If the reduced phototaxis response rate observed in *trxr-1* and *gsr-1* mutant worms is caused by a more oxidative state in these mutants as predicted, then treating these mutant worms with antioxidants shall be able to exert a rescuing effect. To test this, we incubated *trxr-1* and *gsr-1* mutant worms with the antioxidants Vitamin C (VC) and N-acetyl Cysteine (NAC) and then assayed their phototaxis behavior. We found that both VC and NAC greatly potentiated phototaxis response in the two mutants (Fig 5B), supporting the model that the phototaxis defect in *trxr-1* and *gsr-1* mutants may be caused by an oxidative state in these worms. This is also consistent with the model that antioxidants promote LITE-1 function.

### Calcium imaging reveals a defect in the photoresponse in photoreceptor neurons of *trxr-1* and *gsr-1* mutant worms

**Fig 6:**
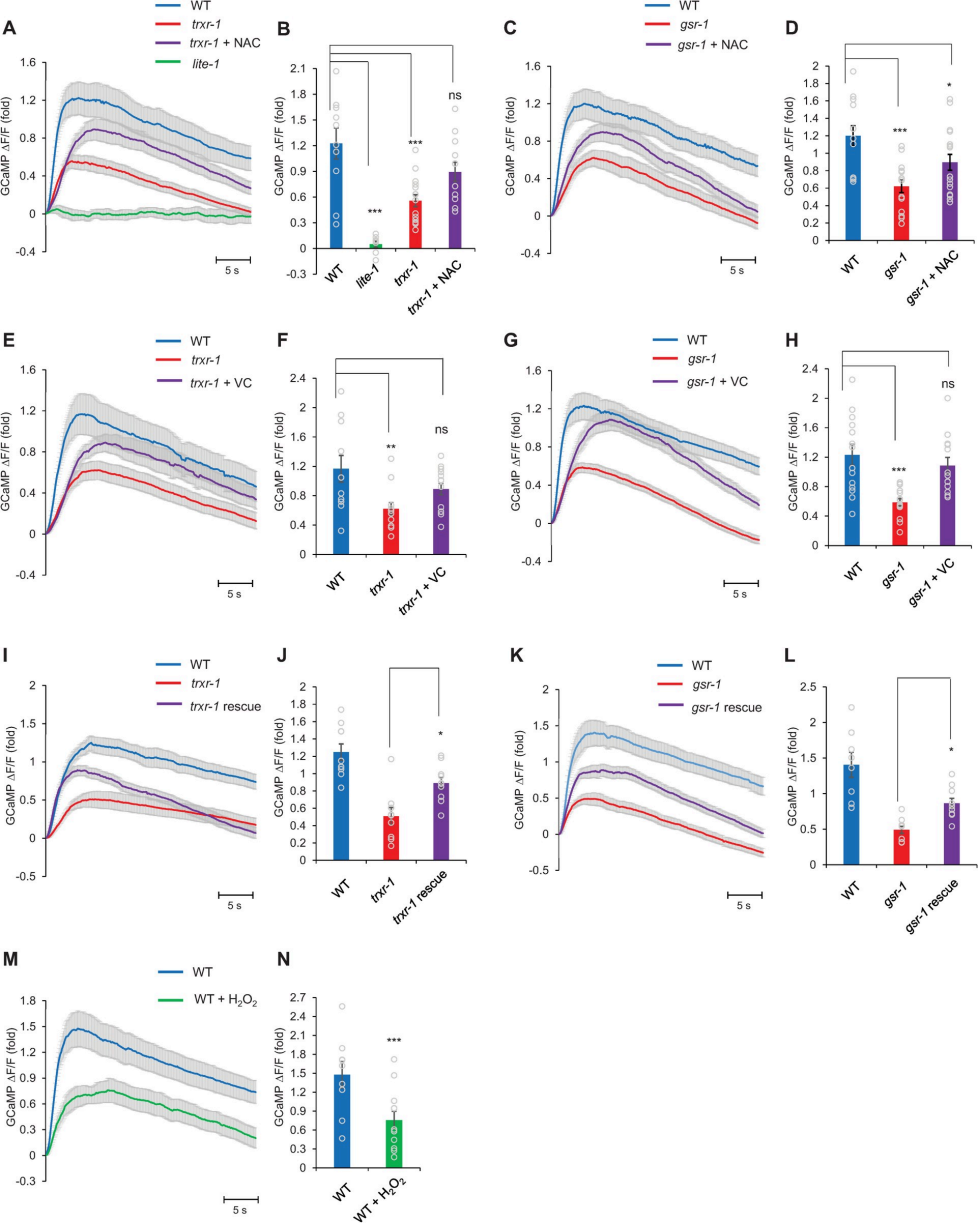
H_2_O_2_ and antioxidants exert opposite effects on the photoresponse in photoreceptor neurons. (A-B) ASH neurons respond to blue wavelength light, and this photoresponse is dependent on LITE-1, consistent with previous report [4]. The photoresponse was reduced in *trxr-1(sv47)* mutant. Pre-treatment with the antioxidant NAC (10 mM) partially rescued the photoresponse defect in *trxr-1* mutant. (A) Average traces with SEM. (B) Error Bars: SEM. n≥8. ***P<0.0005 (ANOVA with Bonferroni test). (C-D) ASH photoresponse was reduced in *gsr-1(xu414)* mutant. Pre-treatment with the antioxidant NAC (10 mM) partially rescued the *gsr-1* mutant photoresponse defect. (C) Average traces with SEM. (D) Error Bars: SEM. n≥12. *P<0.05, ***P<0.0005 (ANOVA with Bonferroni test). (E-F) Pre-treatment with the antioxidant VC (10 mM) partially rescued the photoresponse defect in *trxr-1(sv47)* mutant. (E) Average traces with SEM. (F) Error Bars: SEM. n≥11. **P<0.005(ANOVA with Bonferroni test). (G-H) Pre-treatment with the antioxidant VC (10 mM) partially rescued the photoresponse defect in *gsr-1(xu414)* mutant. (G) Average traces with SEM. (H) Error Bars: SEM. n≥13. ***P<0.0005 (ANOVA with Bonferroni test). (I-J) Transgenic expression of wild type *trxr-1* cDNA in ASH using the *sra-6* promoter partially rescued the *trxr-1* mutant phenotype. (I) Average traces with SEM. (J) Error Bars: SEM. n≥9. *P<0.05 (ANOVA). (K-L) Transgenic expression of wild type *gsr-1* cDNA in ASH using the *sra-6* promoter partially rescued the *gsr-1* mutant phenotype. (K) Average traces with SEM. (L) Error Bars: SEM. n≥8. *P<0.05 (ANOVA). (M-N) Pretreatment with H_2_O_2_ (500 μM) greatly decreased the ASH photoresponse in wild-type worms. (M) Average traces with SEM. N. Error Bars: SEM. n≥7 ***p<0.0005 (t-test).

To further characterize the phenotype of *trxr-1* and *gsr-1* mutants, we went on to record the photoresponse in photoreceptor cells. We first recorded light-induced calcium response in photoreceptor neurons by calcium imaging. We focused on the photoreceptor neuron ASH, as this neuron exhibited a robust photoresponse in calcium imaging assay (Fig 6A and 6B). No photoresponse was detected in *lite-1* mutant worms (Fig 6A and 6B), consistent with previous reports that LITE-1 is the primary photoreceptor in *C*. *elegans* [4–6]. The photoresponse in ASH neurons was reduced in *trxr-1* and *gsr-1* mutants compared to wild-type, suggesting that LITE-1 function is compromised in these mutant worms (Fig 6A and 6B).

By contrast, no notable defect was detected in ASH neurons of *trxr-1* and *gsr-1* mutants in response to octanol (S2B and S2C Fig). In another set of control experiments, we examined odor-evoked (IAA) calcium response in AWC neurons and tastant-evoked (NaCl) calcium response in ASER neuron in both *trxr-1* and *gsr-1* mutant worms, but did not observe a notable defect (S2E, S2F, S2H, and S2I Fig). These control experiments demonstrate that *trxr-1* and *gsr-1* mutant worms do not exhibit a general defect in sensory neurons. Importantly, treating worms with antioxidants such as VC and NAC partially rescued the photoresponse defect in *trxr-1* and *gsr-1* mutants (Fig 6A–6H). A similar rescuing effect was observed in mutant worms expressing wild-type *trxr-1* and *gsr-1* gene as a transgene in ASH neurons (Fig 6I–6L). As expected, both genes were expressed in ASH neurons (S5 Fig). These experiments together demonstrate that *trxr-1* and *gsr-1* mutants exhibit a specific defect in the photoresponse in ASH photoreceptor neurons.

We also examined the effect of H_2_O_2_ on the photoresponse in ASH neurons, and found that H_2_O_2_ treatment greatly decreased the photoresponse in wild-type worms (Fig 6M and 6N). These data reveal that H_2_O_2_ and antioxidants exert opposite effects on the photoresponse in photoreceptor neurons, similar to that observed in phototaxis behavior.

### Patch-clamp recording reveals a defect in the deactivation of the photocurrent in photoreceptor neurons in *trxr-1* and *gsr-1* mutants

While calcium imaging uncovered a defect in the photoresponse in *trxr-1* and *gsr-1* photoreceptor neurons, this assay has some major limitations. As the blue light used to excite the calcium sensor GCaMP also excites photoreceptor neurons (Fig 6), we cannot obtain a baseline of the photoresponse. Nor can we reliably determine the kinetics of the photoresponse, because calcium imaging measures the accumulation of intracellular calcium, which reflects a net outcome of calcium influx, release and clearance. As such, we were only able to reliably quantify the peak calcium level.

**Fig 7:**
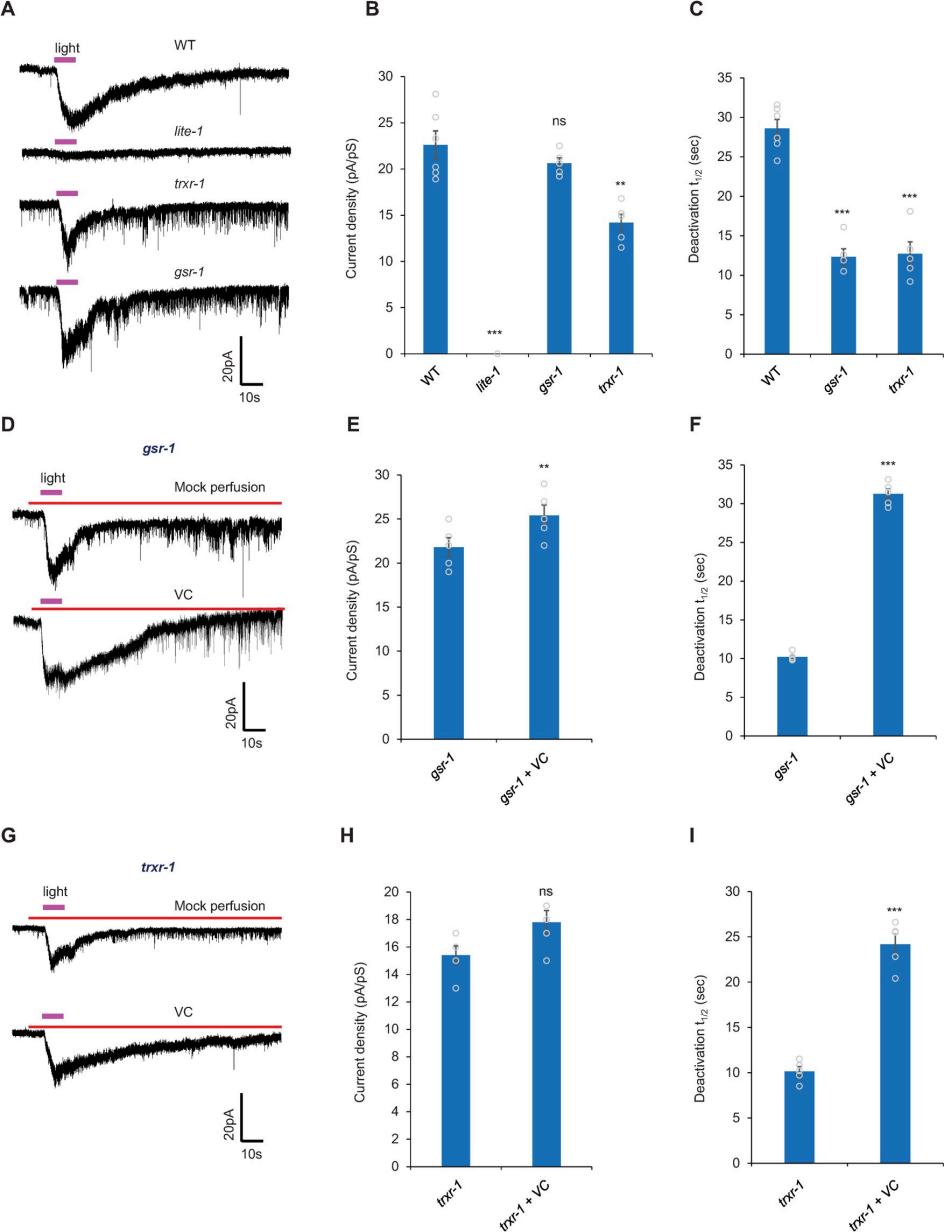
Patch-clamp recording reveals a defect in the deactivation of the photocurrent in photoreceptor neurons in *trxr-1* and *gsr-1* mutants. (A-C) Light-induced conductance in ASH neurons. (A) Sample traces. (B) Bar graph. The density of the photocurrent showed a modest to no reduction in *trxr-1(sv47)* and *gsr-1(xu414)* mutants. Photocurrent was not detected in *lite-1(xu7)* mutants. (C) Bar graph showing that the deactivation of the photocurrent in ASH neuron is accelerated in *trxr-1(sv47)* and *gsr-1(xu414)* mutants compared to WT. (B-C) Error Bars: SEM. n≥5. ***P<0.0005, **P<0.005 (ANOVA with Bonferroni test). Clamping voltage, -60 mV. (D-I) VC (100 μM) perfusion towards ASH neurons immediately prior to light stimulation rescues the photocurrent deactivation defect in *trxr-1(sv47)* and *gsr-1(xu414)* mutants. (D and G) Sample traces. (E and H) Bar graph showing VC application slightly potentiates the amplitude of the photocurrent in *trxr-1(sv47)* (E) and *gsr-1(xu414)* (H) mutants. (F and I) Bar graph showing half-deactivation time t_1/2_ in *trxr-1(sv47)* (F) and *gsr-1(xu414)* (I) mutants. (E-F) Error Bars: SEM. n = 5. **P<0.005, ***P<0.0005 (t-test). (H-I) Error Bars: SEM. n = 5 ***P<0.0005 (t-test).

To overcome the limitations inherent to calcium imaging assays, we recorded light-evoked current in ASH neurons by patch-clamp (Fig 7). No photocurrent was detected in *lite-1* mutant worms (Fig 7A and 7B). This indicates that the photocurrent was mediated by LITE-1, consistent with our behavioral and calcium imaging results, as well as previous electrophysiology data from other photoreceptor neurons [4]. Interestingly, though *trxr-1* and *gsr-1* mutant worms showed a modest or no reduction in the amplitude of the photocurrent in ASH neurons (Fig 7A and 7B), the photocurrent deactivated more rapidly in the two mutants (Fig 7A). Specifically, the deactivation t_1/2_ value showed a great decease in the two mutants (Fig 7C), revealing a severe defect in the deactivation of the photoresponse in *trxr-1* and *gsr-1* mutant worms.

Inspired by our data from behavior and calcium imaging assays, we assayed the effect of antioxidants by perfusing Vitamin C (VC) towards ASH neurons right before light stimulation (Fig 7D and 7G). Though VC only modestly potentiated the amplitude of the photocurrent (Fig 7E and 7H), it greatly slowed down the deactivation of the photocurrent in *trxr-1* and *gsr-1* mutants (Fig 7F and 7I). Thus, antioxidants can rescue the photocurrent deactivation defect in *trxr-1* and *gsr-1* mutant worms.

### H_2_O_2_ inhibits the amplitude of the photocurrent and also facilitates its deactivation

**Fig 8:**
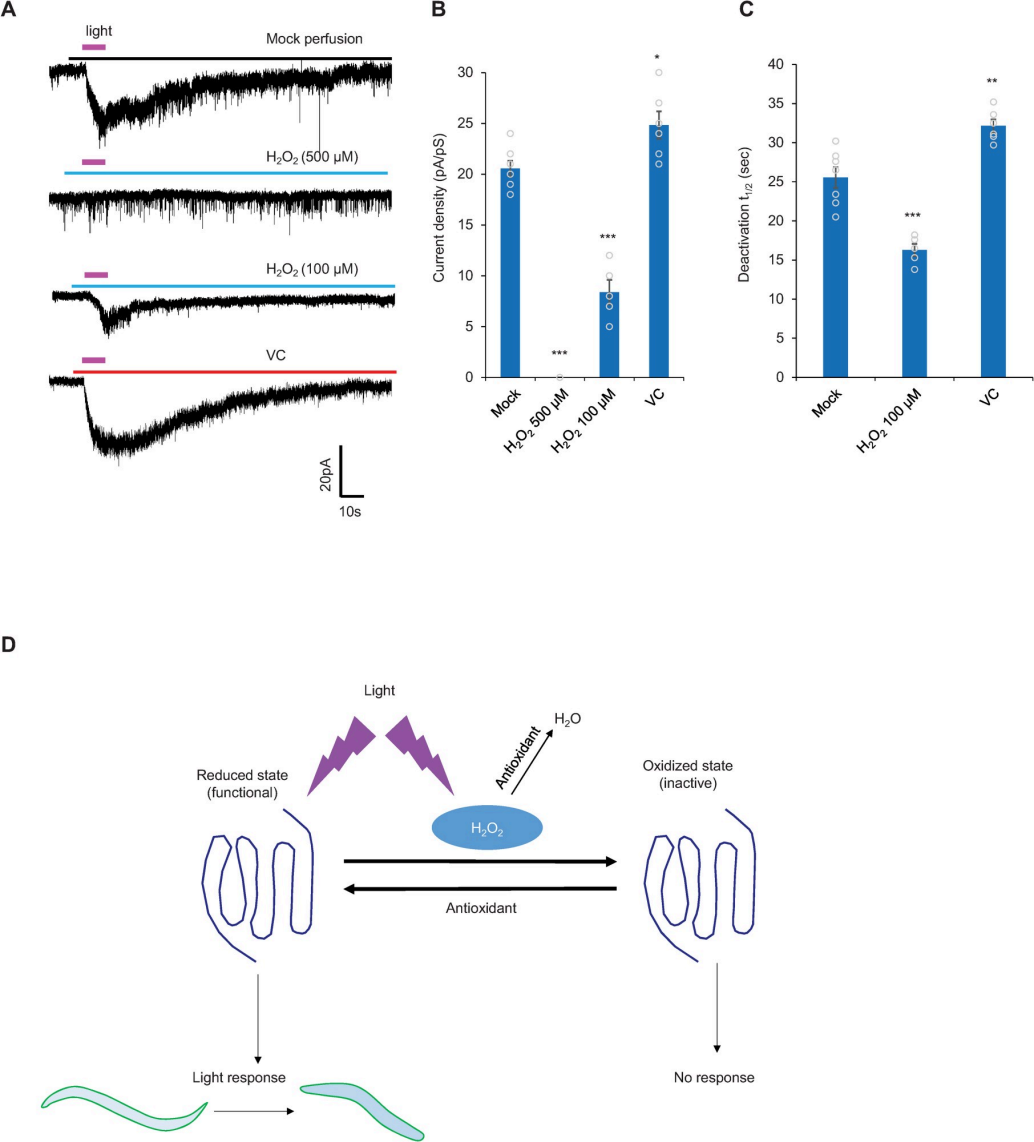
H_2_O_2_ and antioxidants act reciprocally to deactivate and potentiate the photocurrent, respectively. (A-C) H_2_O_2_ inhibits the amplitude of the photocurrent and also facilitates its deactivation. Conversely, the antioxidant VC (100 μM) potentiates the amplitude of the photocurrent and reduces the rate of the deactivation. (A) Sample traces. (B) Bar graphs showing the peak current density (pA/pS) for each treatment. (C) Bar graph showing the rate of the deactivation of the photocurrent for each treatment. (B-C) Error Bars: SEM. n≥5 ***p<0.0005, **p<0.005, *p<0.05 (ANOVA with Bonferroni test). (D) Model showing how LITE-1 is regulated by H_2_O_2_ and antioxidants. As UV light illumination generates ROS such as H_2_O_2_ [7], we propose that upon light activation of LITE-1, H_2_O_2_ produced by light then deactivates LITE-1, providing a novel mechanism to terminate the photoresponse. Antioxidants may promote the reduction of oxidized LITE-1 to facilitate its recovery.

The defect in photoresponse deactivation in *trxr-1* and *gsr-1* mutant worms and its rescue by antioxidants suggest that H_2_O_2_ may inhibit the photocurrent. To test this, we perfused ASH neurons with H_2_O_2_ right before light stimulation, and found that H_2_O_2_ (500 μM) completely blocked the photocurrent (Fig 8A and 8B). We also tested a lower concentration of H_2_O_2_ (100 μM), and found that it blocked the photocurrent by ~60% (Fig 8A and 8B). This allowed us to quantify the deactivation of the photocurrent remaining in H_2_O_2_-treated worms. Indeed, compared to mock-treated control, the residual photocurrent in H_2_O_2_-treated ASH neurons deactivated more rapidly with a significantly reduced t_1/2_ value (Fig 8C). Thus, H_2_O_2_ not only inhibits the amplitude of the photocurrent, but also accelerates the deactivation of the photocurrent.

To gather additional evidence, we performed the converse experiment by testing the effect of antioxidants on the photocurrent (Fig 8A). Perfusing the antioxidant Vitamin C (VC) towards ASH neurons right before light illumination not only potentiated the amplitude of the photocurrent (Fig 8A and 8B), but also reduced the rate of the deactivation of the photocurrent (Fig 8C). This provides further evidence supporting that H_2_O_2_ not only inhibits the amplitude of the photocurrent, but also accelerates its deactivation. As UV light illumination is known to generate ROS such as H_2_O_2_ [7], we propose that upon light activation of LITE-1, H_2_O_2_ produced by light then deactivates LITE-1, providing a potential mechanism to terminate the photoresponse (Fig 8D).

## Discussion

LITE-1 represents a unique type of photoreceptor that mediates photosensation in *C*. *elegans* [5]. However, it is unclear how LITE-1 is regulated. Here, we conducted an unbiased genetic screen for mutants that suppress LITE-1 function. One major group of *lite-1* suppressor genes encode proteins involved in regenerating thioredoxin and glutathione, the two major antioxidants in the cell [9], suggesting that reduction-oxidation regulates LITE-1 function. Through a combination of behavioral, calcium imaging and electrophysiological analyses, we show that H_2_O_2_ inhibits LITE-1-mediated photoresponse, while antioxidants promote it. Interestingly, although other sensory functions can also be impaired by H_2_O_2_, photosensation appears to be more vulnerable to this oxidant, revealing a relatively higher sensitivity of photosensation to the redox state. It remains possible that H_2_O_2_ may inhibit LITE-1 function indirectly through another component in the phototransduction pathway. Nevertheless, given previous biochemical data showing that H_2_O_2_ directly inhibits LITE-1 function *in vitro* [5], we suggest that LITE-1 may also be a H_2_O_2_ target *in vivo*. One potential substrate of H_2_O_2_ is the two tryptophan residues in LITE-1 that are crucial for light absorption [5], though other possibilities remain. UV light illumination is well known to produce H_2_O_2_ [7]. We thus propose that following light activation of LITE-1, H_2_O_2_ resulting from light illumination then deactivates LITE-1, providing a potential mechanism to terminate the photoresponse (Fig 8D).

In addition to H_2_O_2_, our results also unveil an important role of the antioxidants thioredoxin and glutathione in regulating LITE-1. Following the deactivation of LITE-1, the oxidized LITE-1 would need to recover from this inactive state. Antioxidants, particularly thioredoxin, can act as reductases to facilitate the reduction of oxidized proteins [10,11]. Indeed, we found that *trxr-1* and *gsr-1* mutants are defective in recovering from light-induced photo-insensitivity (Fig 4B and 4D). We propose that upon LITE-1 deactivation by H_2_O_2_, antioxidants may promote the recovery of LITE-1 from its inactive state (Fig 8D). Our studies thus provide a potential mechanism by which oxidants and antioxidants act synergistically to regulate photosensation.

As an oxidizing agent, H_2_O_2_ has the potential to non-specifically damage all proteins, lipids and nucleic acids, and is generally not considered playing specific roles in regulating cellular functions. However, an increasingly growing body of evidence demonstrates that H_2_O_2_ can target selective proteins to specifically regulate a plethora of physiological processes particularly at low concentrations, ranging from neuronal signaling to aging and longevity [16–19], though at high concentrations, the nonspecific effect of H_2_O_2_ may dominate. It is currently unclear why photosensation is more vulnerable to H_2_O_2_ than other tested sensory functions. One possibility is that LITE-1 is a more sensitive target of H_2_O_2_ than other sensory receptors, given that LITE-1 can be readily inhibited by H_2_O_2_ *in vitro* [5]. Future studies are needed to test this and determine the underlying biochemical mechanisms. It should also be noted that since the suppressor screen was conducted on worms ectopically expressing LITE-1 in body-wall muscles, the genes isolated from this screen might not necessarily regulate LITE-1 in its native environment. Despite this caveat, we found that at least in ASH photoreceptor neurons, *trxr-1* and *gsr-1*, as well as H_2_O_2_ and antioxidants, all affect LITE-1 function.

LITE-1 is a member of the invertebrate gustatory receptor (GR) family, which was first identified in *Drosophila* [20,21]. Five GR members are found in the worm genome [4]. Interestingly, another GR member GUR-3 can also be activated by UV light but through a distinct mechanism [7]. Specifically, H_2_O_2_ produced by light illumination activates GUR-3 [7]. In this case, GUR-3 functions as a H_2_O_2_ sensor but not a light sensor [7]. It is striking that two closely related GR members are differentially regulated by H_2_O_2_, with one inhibited by H_2_O_2_ (i.e. LITE-1) and the other activated by H_2_O_2_ (i.e. GUR-3). Future studies are needed to elucidate the detailed mechanisms underlying this differential regulation by H_2_O_2_.

Thioredoxin and glutathione are the two primary antioxidants produced by the cell, which help the cell maintain a reducing environment [9,10]. In addition, similar to the case with H_2_O_2_, these two antioxidants, particularly thioredoxin, play specific roles in regulating cellular signaling through target proteins [13]. It is notable that our suppressor screen only identified genes involved in the regeneration but not the biosynthesis of these two antioxidants. In the case of glutathione, perhaps its loss is detrimental to the worm, as our screen purposely excluded the mutants that were rather unhealthy and uncoordinated. On the other hand, as at least five genes encode thioredoxin in the worm genome, the potential functional redundancy may explain the lack of thioredoxin mutants from our screen. Functional redundancy may also explain the fact that we did not isolate any mutants lacking glutathione peroxidase (Gpx) or peroxiredoxin (Prx), both of which directly clear H_2_O_2_ [9,10] (Fig 2B). Another interesting observation is that we only isolated *trxr-1* but not *trxr-2* mutants. A major distinction between these two enzymes is that the former acts in the cytosol while the latter functions in the mitochondria [9]. It is possible that LITE-1 is more sensitive to the redox state in the cytosol than that in the mitochondria.

Timely deactivation of the photoresponse is an important process in phototransduction, as it allows the photoreceptor to respond to subsequent light stimuli [1,22–24]. In addition, as an avoidance response, it would be beneficial for the worm to terminate phototaxis behavior in a timely manner once it has escaped the light environment. For example, it would not be energy efficient for the worm to constantly maintain light-evoked behavioral responses (reversals and high locomotion speed) after the cessation of the light stimulus. It might be argued that a rapid inactivation of LITE-1 would not offer sufficient time for the worm to escape the light environment. This, however, should not be a concern, as the downstream phototransduction pathway would remain active for some time to maintain the activity of photoreceptor cells as well as the downstream neural circuits that drive the escape behavior. The deactivation of the photoresponse is a complex process in vertebrate and insect photoreceptor cells, which occurs at multiple levels, including the photoreceptor, G protein, and downstream signaling components [1,22–24]. For example, at the photoreceptor level, light-activated photoreceptor such as rhodopsin is phosphorylated by a rhodopsin kinase (GRK), which recruits arrestin [1,22–24]. This prevents rhodopsin from activating G protein, thereby shutting down rhodopsin [1,22–24]. As LITE-1 adopts a reversed membrane topology [5], it may not bind to arrestin, suggesting that LITE-1 is probably not regulated by this mechanism. Our findings suggest that H_2_O_2_ produced by light illumination inhibits the photoreceptor. This points to an interesting model that light itself can deactivate the photoreceptor to terminate the photoresponse (Fig 8D).

## Materials and methods

### Strains, genetics and molecular biology

Wild type: N2. TQ800: *lite-1(xu7)*. TQ6680: *xuIs98[Pmyo-3*::*lite-1(L)*::*SL2*::*YFP]*. TQ6936: *xu407*. TQ6934: *xu408*. TQ6938: *xu409*. TQ7006: *xu413*. TQ6929: *xu414*. TQ6937: *xu415*. TQ6926: *xu416*. TQ6931: *xu418*. TQ6927: *xu419*. TQ6940: *xu420*. TQ1376a: *xu421*. TQ9984: *trxr-1(sv47); xuEx3397[Pmyo-3*::*trxr-1*::*SL2*::*CFP]; xuIs98[Pmyo-3*::*lite-1(L)*::*SL2*::*YFP]*. TQ9949: *gsr-1(xu414); xuEx3398[Pmyo-3*::*gsr-1*::*SL2*::*CFP]; xuIs98[Pmyo-3*::*lite-1(L)*::*SL2*::*YFP]*. TQ9803: *trxr-1(sv47)* x5 outcrossed. TQ9854: *trxr-1(sv47); xuIs98[Pmyo-3*::*lite-1(L)*::*SL2*::*YFP]*. TQ9802: *gsr-1(xu414)*. TQ5856: *xuEx1978[Psra-6*::*Gcamp6+Psra-6*::*sl2*::*DsRed]*. TQ10019: *trxr-1(sv47); xuEx1978[Psra-6*::*GCamp6+Psra-6*::*sl2*::*DsRed]*. TQ9855: *gsr-1(xu414); xuEx1978[Psra-6*::*Gcamp6+Psra-6*::*sl2*::*DsRed]*. TQ1764: *xuEx631[Psra-6*::*DsRed + Pstr-3*::*yfp2]*. TQ9853: *trxr-1(sv47); xuEx631[Psra-6*::*DsRed + Pstr-3*::*yfp2]*. TQ10020: *gsr-1(xu414); xuEx631[Psra-6*::*DsRed + Pstr-3*::*yfp2]*. TQ6435: *lite-1(xu7); xuEx631[Psra-6*::*DsRed + Pstr-3*::*yfp2]*. TQ8220: *lite-1(xu492); xuEx1978[Psra-6*::*GCamp6+Psra-6*::*sl2*::*DsRed]*. TQ7189: *xuEx2631[Pstr-2*::*GCaMP6(f)+Podr-1*::*DsRed]*. TQ10136: *gsr-1(xu414); xuEx2631[Pstr-2*::*GCaMP6(f)+Podr-1*::*DsRed]*. TQ10137: *trxr-1(sv47); xuEx2631[Pstr-2*::*GCaMP6(f)+Podr-1*::*DsRed]*. TQ86: *tax-4(p678)*. TQ10188: *gsr-1(xu414); xuEx3455[Psra-6*::*gsr-1 cDNA*::*SL2*::*CFP];xuEx1978[Psra-6*::*GCaMP+Psra-6*::*DsRed]*. TQ10189: *trxr-1(sv47); xuEx3456[Psra-6*::*trxr-1 cDNA PCR product+Punc-122*::*GFP];xuEx1978[Psra-6*::*GCaMP+Psra-6*::*DsRed]*. TQ10168: *trxr-1(sv47); prdx-2(gk169); xuIs98[Pmyo-3*::*lite-1(L)*::*SL2*::*YFP]*. *TQ8078*: *xuEx3000[Pgcy-5*::*GCaMP6(f)]*. TQ9273: *tax-4(p678); xuEx3000[Pgcy-5*::*GCaMP6(f)]*. TQ10165: *trxr-1(sv47); xuEx3000[Pgcy-5*::*GCaMP6(f)]*. TQ10166: *gsr-1(xu414); xuEx3000[Pgcy-5*::*GCaMP6(f)]*. TQ3104a: *trxr-1(sv47); xuEx315a[Pmyo-3*::*gsr-1 RNAi(S+AS)*::*SL2*::*mcherry2]; xuIs98[Pmyo-3*::*lite-1(L)*::*SL2*::*YFP]*. TQ3107a: *gsr-1(xu414); xuEx317a[Pmyo-3*::*trxr-1 RNAi(S+AS)*::*SL2*::*mcherry2]; xuIs98[Pmyo-3*::*lite-1(L)*::*SL2*::*YFP]*. TQ3106a: *xuEx310a[Psra-6*::*SL2*::*mcherry2];xuEx309a[Ptrxr-1*::*SL2*::*YFP]*. TQ3108b: *xuEx310a[Psra-6*::*SL2*::*mcherry2];xuEx312a[Pgsr-1*::*gsr-1*::*SL2*::*YFP]*. TQ472: *osm-9(ky10)*. TQ2814a: *osm-9(ky10); xuEx1978[Psra-6*::*GCaMP+Psra-6*::*DsRed]*.

Worms were cultured at 20°C on standard nematode growth medium (NGM). *trxr-1* and *gsr-1* cDNA were cloned by RT-PCR from total RNA isolated from WT (N2) worms. Expression of the transgene was verified by CFP expression, which was driven by SL2 from the same transcript.

### Genetic screen

EMS was used to mutagenize worms ectopically expressing *lite-1* in the body-wall muscle (*xuIs98[Pmyo-3*::*lite-1(L)*::*SL2*::*YFP])* [5]. F2 progenies were placed in 96-well plates containing 50 μl M9, with 5–6 individuals placed per well. UV-A light (360 ± 20 nm, -1.52 log I/I_o_) was used to illuminate individual wells for 30 sec, and candidates that failed to paralyze during this exposure were recovered for subsequent analysis. I_o_ was set as 20 mW in this study. As previously described [25], candidates were outcrossed to the parental strain at least five times, and both the parental and candidate strains were subjected to whole-genome sequencing (WGS). Analysis of the sequencing data was performed as previously described [25,26]. By comparing the WGS data between parental and mutant strains, we obtained density maps for each candidate and mapped the mutations in 11 candidate strains.

### Behavioral assays

The light-induced paralysis assay was performed on candidate mutants carrying *xuIs98[Pmyo-3*::*lite-1(L)*::*SL2*::*YFP]* with blue wavelength light [480 ± 20 nm, -0.05 log I/I_o_]. Time to paralyze upon light exposure was recorded manually, with a maximum time of 30 sec. Each worm was tested once, with a total of 30 worms tested per genotype. Average time to paralyze was calculated for each genotype.

Phototaxis response was tested as previously described [3]. Briefly, day 1 adult hermaphrodite worms were transferred individually to NGM plates containing a thin lawn of freshly seeded OP50 bacteria, followed by a 10-minute recovery period to stabilize behavior. For all phototaxis experiments described, UV-A light [360 ± 20 nm, -1.52 log I/I_o_] was used. To examine the phototaxis response, a 2 sec UV-A light pulse was focused on the head of the worm using a 10x objective with a 6.4x zoom lens on a Zeiss microscope (Zeiss Discovery). A response was scored if the worm reversed within three sec of cessation of light stimulus with a backward movement comprising at least half of a head swing. Each worm was tested five times with an 8–10 minute interval between each trial, and the percent response for each individual was determined. Background light used to visualize worms was filtered into red using a red filter.

Pre-exposure experiments were performed by exposing worms to a UV-A light pulse for varying durations (0, 5, 10, 15, or 20 sec), followed by a five-second rest period prior to performing the phototaxis assay as described above. For pre-exposure experiments, each worm was tested once, with the response rate of 5–10 individuals averaged and counted as a single trial, and repeated for a total of 10 trials per group.

For recovery experiments, worms were pre-exposed to UV-A light for 25 sec, followed by a recovery period at various time points (0, 15, 30, 45, 60, 75, or 90 sec), after which the phototaxis assay was performed. Each worm was tested once, with the response rate of 5–10 individuals averaged and counted as a single trial, and repeated for a total of 10 trials per group.

For pre-treatment experiments, day 1 worms were collected and transferred into an microcentrifuge tube containing 1 ml of M13 buffer [30mM Tris Base, 100mM NaCl, 10mM KCl, pH 7] with concentrated OP50 to prevent starvation, as well as the specified treatment condition: Mock (1× M13), H_2_O_2_ (500 μM), Vitamin C (10 mM), N-acetyl cysteine (10 mM). Worms were incubated for 2 hours at 20°C. Following the incubation period, worms were recovered to NGM plates, allowed to fully recover for 10–20 minutes, and tested for the phototaxis response. For antioxidant-treated animals, the phototaxis assay was performed as described in Fig 4A and 4C, using 15 sec of light pre-exposure period followed by a 5 sec recovery before performing the phototaxis assay.

Acute avoidance to octanol odor was modified from previously described methods [27]. Briefly, animals were tested on standard NGM plates dried for one hour without lids to remove any excess surface moisture. Prior to testing, plates were seeded with a thin lawn of fresh OP50 in the center of the plate to prevent animals from leaving, which was allowed to dry for 10 minutes with the lid off prior to placing animals on the plate. Three Day 1 adult worms were transferred to the testing plate and allowed to habituate for 10 minutes prior to testing. Octanol odor avoidance was then assessed using a glass needle containing 100% octanol placed in front of a forward moving animal, with the time to respond recorded using a stopwatch. Pre-treatment experiments with H_2_O_2_ were performed as described above prior to testing. For all octanol avoidance assays, each worm was tested five times to determine the average time to respond.

Chemotaxis assays were modified from previously described methods [28]. Test plates for chemotaxis were made using 10 cm diameter assay plates (5 mM KPO4, pH 6.0, 1 mM CaCl2, 1mM MgSO4, 2% agar). Well-fed Day 1 adult animals were collected, washed, and transferred with chemotaxis buffer (25 mM potassium phosphate (pH 6.0), 1 mM CaCl2, 1 mM MgSO4) two times followed by a final wash in S-basal ((5.85 g NaCl, 1 g K2HPO4, 6g KH2PO4, 1 mL cholesterol (5 mg/ml in ethanol), H2O to 1L). Fifty to two hundred animals were tested for each plate. Chemoattraction to the odorant isoamyl alcohol (IAA) was performed by placing worms near the center of the plate at a point equidistant to the point source attractant (1% IAA diluted in ethanol) and the counterattractant (ethanol). Sodium azide (500 mM, two microliters) was also spotted at the attractant and counterattractant points just prior to testing to paralyze worms that reached the point sources. After placing, the assay ran for one hour at 20°C. The chemotaxis index (C.I.) was calculated as described previously [28]. For the salt chemotaxis assay, a salt gradient was formed overnight on the chemotaxis assay plate by placing a 2% agar plug (10 mm diameter) made in chemotaxis buffer containing 100 mM of NaCl. Fifty to two hundred animals were tested for each plate. Two microliters of 500 mM sodium azide were allowed to absorb at the gradient peak and the opposite end of the plate just prior to placing animals in a central start box. After placing, the assay ran for one hour at 20°C. The chemotaxis index (C.I.) was calculated as described previously [29]. Chemotaxis assays were repeated at least 10 times for each experimental condition.

### Calcium imaging

Calcium imaging was performed on an Olympus IX73 inverted microscope under a 40× objective. Images were acquired using an ORCA-Flash 4.0 sCMOS camera (Hammatsu Inc.) with MetaFluor software (Molecular Devices Inc.). To image the photoresponse in ASH neuron, we used a microfluidic system as described previously [30]. Briefly, young adult worms were loaded into the chip mounted on the microscope and incubated with 1× M13 buffer [30 mM Tris, 100 mM NaCl, 10 mM KCl, pH 7] throughout analysis. Worms were exposed to blue wavelength light [480 ± 20 nm, -0.89 log I/I_o_] for 30 sec while recording GCaMP fluorescent intensity. Peak percentage change in the intensity of GCaMP fluorescence in response to light was scored. Pre-treatment exposure with H_2_O_2_, VC, or NAC was performed as described for the behavioral assays prior to calcium imaging.

Calcium imaging of neuronal responses to 0.2% octanol, 0.01% IAA, or 50 mM NaCl were performed using a microfluidic system as previously described [30]. Briefly, day 1 worms were loaded into the chip mounted on the microscope, and treatment was given by switching solutions administered to the nose tip. Due to the intrinsic light-sensitivity of ASH neurons [3], we pre-exposed worms to light for 2 minutes to establish a baseline before administering the stimulation. Octanol and IAA were diluted in M13 buffer [30 mM Tris, 100 mM NaCl, 10 mM KCl, pH 7]. 50 to 0 mM salt concentration changes were performed by switching solutions administered to the nose tip (25 mM potassium phosphate [pH 6.0], 1 mM CaCl2, 1 mM MgSO4, 0.02% gelatin, and either 50 or 0 mM NaCl with glycerol to adjust osmolarity to 350 mOsm). Sample size was greater than or equal to 10 for each genotype.

### Electrophysiology

Whole-cell patch-clamp recording on worm ASH neuron was performed as previously described [30]. Briefly, recordings were performed on an Olympus upright microscope with an EPC-10 amplifier under a 60x objective. Worms were glued and dissected on a sylgard-coated coverglass to expose ASH neurons. Standard bath solution was as follows (in mM): 140 NaCl, 2.5 KCl, 1 CaCl_2_, 1 MgCl_2_, 20 glucose and 5 HEPES (320 mOsm; pH adjusted to 7.3). Pipette solution: 115 K-gluconate, 15 KCl, 1 MgCl_2_, 10 HEPES, 0.25 CaCl_2_, 20 sucrose, 5 BAPTA, 5 Na_2_ATP and 0.5 NaGTP (315 mOsm; pH adjusted to 7.2). To identify ASH neurons for recording, we labeled ASH and ASI with fluorescent markers to aid in dissection by using the transgenic line *xuEx631[Psra-6*::*DsRed + Pstr-3*::*yfp2]*. Clamping voltage: −60 mV. Both series resistance and membrane capacitance were compensated during recording. Background light was filtered red. A 10 sec pulse of UV-A light [360 ± 20 nm; −1.75 log I/I_o_] was used to evoke photocurrents in ASH neurons. To measure light intensity, we used a radiometric ultraviolet-specific sensor head (268S, UDT Instruments) coupled to an optometer (S471, UDT Instruments). UV light stimulus was administered to neurons for 10 sec from an Arc lamp (EXFO) controlled by a mechanical shutter (Sutter) triggered by the amplifier. For perfusion experiments, we perfused H_2_O_2_ (100 μM or 500 μM) or VC (100 μM) in the chamber.

### Statistics and data analysis

Statistical analysis was performed in GraphPad Prism.

## Supporting information

S1 Fig Loss of *prdx-2* does not affect the *trxr-1* defect. (A) *trxr-1(sv47); prdx-2(gk169)* double mutant showed a similar defect to *trxr-1(sv47)* single mutant in LITE-1-dependent light-evoked paralysis assay. Error Bars: SEM. n≥30 ***p<0.0005(ANOVA with Bonferroni test).
(PDF)

S2 Fig *trxr-1* and *gsr-1* mutants show normal behavioral responses to octanol, IAA and NaCl. (A) *trxr-1(sv47)* and *gsr-1(xu414)* mutant worms showed normal octanol avoidance behavior. As a control, *osm-9(ky10)* were found to be defective in this behavior. Error Bars: SEM. n≥30 ***p<0.0005 (ANOVA with Bonferroni test). (B-C) Calcium imaging shows that *trxr-1(sv47)* and *gsr-1(xu414)* mutant worms exhibit normal calcium response to octanol compared to WT, while as a control, *osm-9 (ky10)* were defective in this response. (B) Average traces with SEM. (C) Error Bars: SEM. n≥10. ***p<0.0005 (ANOVA with Bonferroni test). (D) *trxr-1(sv47)* and *gsr-1(xu414)* mutant worms showed normal chemotaxis to IAA. As a control, *tax-4(p678)* were found to be defective in this behavior. Error Bars: SEM. n = 13 ***p<0.0005 (ANOVA with Bonferroni test). (E-F) Calcium imaging shows that *trxr-1(sv47)* and *gsr-1(xu414)* mutant worms exhibit normal calcium response to IAA compared to WT, while as a control, *tax-4 (p678)* were defective in this response. (E) Average traces with SEM. (F) Error Bars: SEM. n≥10. (G) *trxr-1(sv47)* and *gsr-1(xu414)* mutant worms showed normal salt chemotaxis behavior. As a control, *tax-4(p678)* worms were found to be defective in this behavior. Error Bars: SEM. n = 10 ***p<0.0005 (ANOVA with Bonferroni test). (H-I) Calcium imaging shows that *trxr-1(sv47)* and *gsr-1(xu414)* mutant worms display normal calcium response to IAA compared to WT, while as a control, *tax-4 (p678)* were defective in this response. (H) Average traces with SEM. (I) Error Bars: SEM. n≥10. ***p<0.0005 (ANOVA with Bonferroni test).
(PDF)

S3 Fig Paraquat mildly inhibits phototaxis behavior. (A) Worms were pretreated with paraquat (0.5 mM) for 2 hours and then tested for phototaxis response. Error Bars: SEM. n = 10. *p<0.05 (t-test).
(PDF)

S4 Fig The effect of H_2_O_2_ on octanol and osmotic avoidance behaviors. (A) Low concentrations of H_2_O_2_ do not affect octanol avoidance while high concentrations of H_2_O_2_ impair it. Wild-type worms were treated with 0.5 mM and 5 mM H_2_O_2_ for 2 hours and then tested for their avoidance response to 100% octanol. The latency time taken by the worms to respond to octanol was quanitied. Error Bars: SEM. n≥30. ***p<0.0005 (ANOVA with Bonferroni test). (B) Low concentrations of H_2_O_2_ do not affect osmotic avoidance behavior while high concentrations of H_2_O_2_ impair it. Wild-type worms were treated with 0.5 mM and 5 mM H_2_O_2_ for 2 hours and then tested for their avoidance response to 0.4 M glycerol. The percent of the worms responding to glycerol was quantified. Error Bars: SEM. n≥30. ***p<0.0005 (ANOVA with Bonferroni test).
(PDF)

S5 Fig Expression patterns of *trxr-1* and *gsr-1*. (A) *trxr-1* is expressed in ASH neurons. A genomic fragment (757 bp promoter region) of *trxr-1* gene was used to drive the expression of YFP. *Psra-6*::*SL2*::*mCherry* was used to mark ASH neurons. Shown are confocal images. The arrows point to ASH. (B) *gsr-1* is expressed in ASH neurons. A ~2.3 kb genomic fragment of *gsr-1* gene (324 bp promoter and the entire coding region) was used to drive the expression of YFP. *Psra-6*::*SL2*::*mCherry* was used to mark ASH neurons. Shown are confocal images. The arrows point to ASH.
(PDF)
