# Supplementary figures and images for: Regulation of photosensation by hydrogen peroxide and antioxidants in C. elegans
Source: PLoS Genet. 2020 Dec 10;16(12):e1009257. doi: 10.1371/journal.pgen.1009257 (PMC7755287; doi:10.1371/journal.pgen.1009257)

Figure S1

A

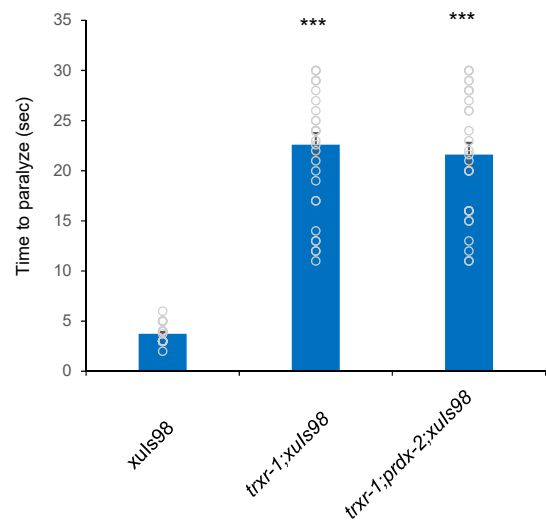

Supplement: S1 Fig — (A) trxr-1(sv47); prdx-2(gk169) double mutant showed a similar defect to trxr-1(sv47) single mutant in LITE-1-dependent light-evoked paralysis assay. Error Bars: SEM. n≥30 ***p<0.0005(ANOVA with Bonferroni test). (PDF) [file pgen.1009257.s001.pdf]

**Figure S2**

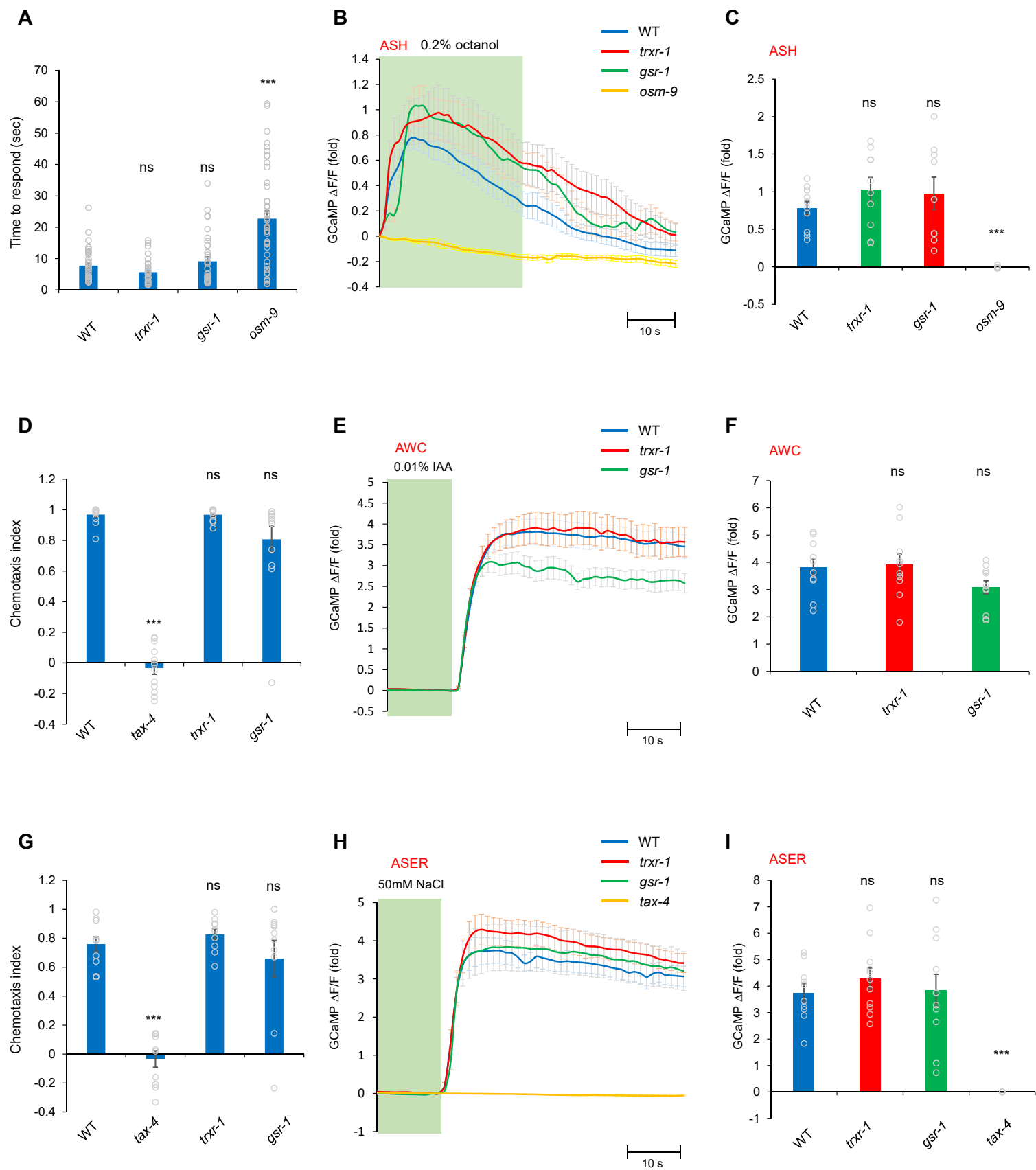

Supplement: S2 Fig — (A) trxr-1(sv47) and gsr-1(xu414) mutant worms showed normal octanol avoidance behavior. As a control, osm-9(ky10) were found to be defective in this behavior. Error Bars: SEM. n≥30 ***p<0.0005 (ANOVA with Bonferroni test). (B-C) Calcium imaging shows that trxr-1(sv47) and gsr-1(xu414) mutant worms exhibit normal calcium response to octanol compared to WT, while as a control, osm-9 (ky10) were defective in this response. (B) Average traces with SEM. (C) Error Bars: SEM. n≥10. ***p<0.0005 (ANOVA with Bonferroni test). (D) trxr-1(sv47) and gsr-1(xu414) mutant worms showed normal chemotaxis to IAA. As a control, tax-4(p678) were found to be defective in this behavior. Error Bars: SEM. n = 13 ***p<0.0005 (ANOVA with Bonferroni test). (E-F) Calcium imaging shows that trxr-1(sv47) and gsr-1(xu414) mutant worms exhibit normal calcium response to IAA compared to WT, while as a control, tax-4 (p678) were defective in this response. (E) Average traces with SEM. (F) Error Bars: SEM. n≥10. (G) trxr-1(sv47) and gsr-1(xu414) mutant worms showed normal salt chemotaxis behavior. As a control, tax-4(p678) worms were found to be defective in this behavior. Error Bars: SEM. n = 10 ***p<0.0005 (ANOVA with Bonferroni test). (H-I) Calcium imaging shows that trxr-1(sv47) and gsr-1(xu414) mutant worms display normal calcium response to IAA compared to WT, while as a control, tax-4 (p678) were defective in this response. (H) Average traces with SEM. (I) Error Bars: SEM. n≥10. ***p<0.0005 (ANOVA with Bonferroni test). (PDF) [file pgen.1009257.s002.pdf]

Figure S3

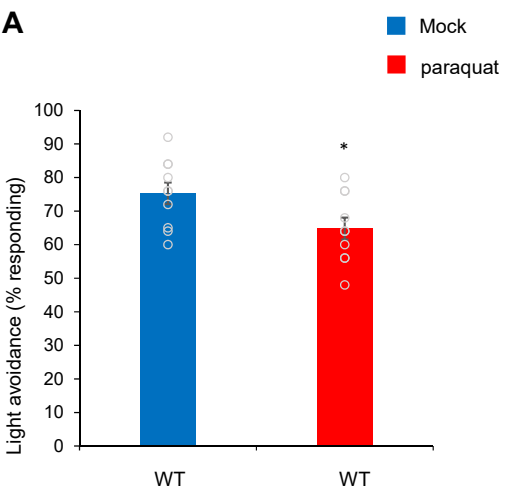

Supplement: S3 Fig — (A) Worms were pretreated with paraquat (0.5 mM) for 2 hours and then tested for phototaxis response. Error Bars: SEM. n = 10. *p<0.05 (t-test). (PDF) [file pgen.1009257.s003.pdf]

Figure S4

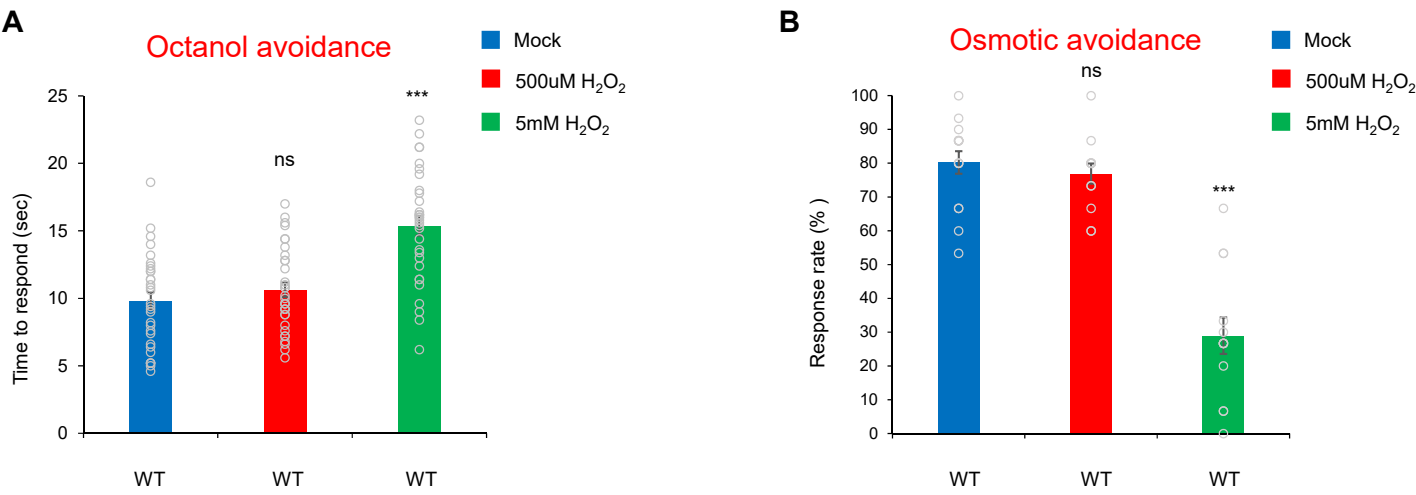

Supplement: S4 Fig — (A) Low concentrations of H2O2 do not affect octanol avoidance while high concentrations of H2O2 impair it. Wild-type worms were treated with 0.5 mM and 5 mM H2O2 for 2 hours and then tested for their avoidance response to 100% octanol. The latency time taken by the worms to respond to octanol was quanitied. Error Bars: SEM. n≥30. ***p<0.0005 (ANOVA with Bonferroni test). (B) Low concentrations of H2O2 do not affect osmotic avoidance behavior while high concentrations of H2O2 impair it. Wild-type worms were treated with 0.5 mM and 5 mM H2O2 for 2 hours and then tested for their avoidance response to 0.4 M glycerol. The percent of the worms responding to glycerol was quantified. Error Bars: SEM. n≥30. ***p<0.0005 (ANOVA with Bonferroni test). (PDF) [file pgen.1009257.s004.pdf]

Figure S5

A

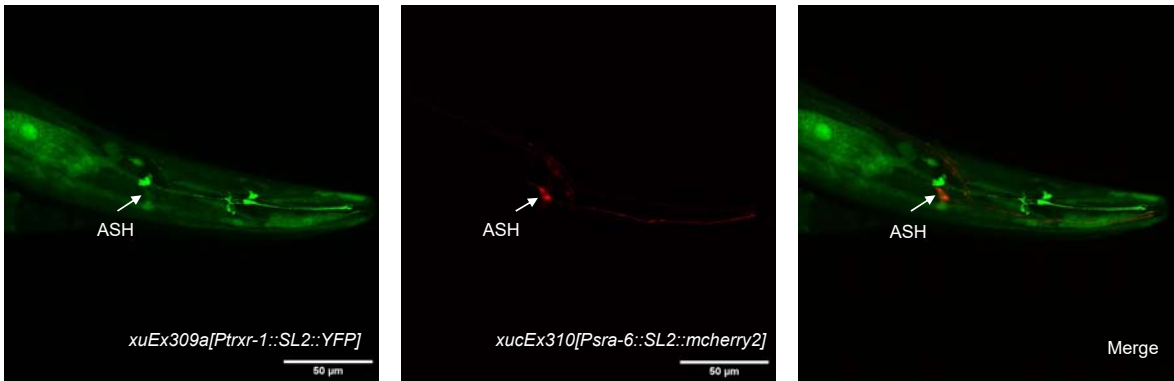

B

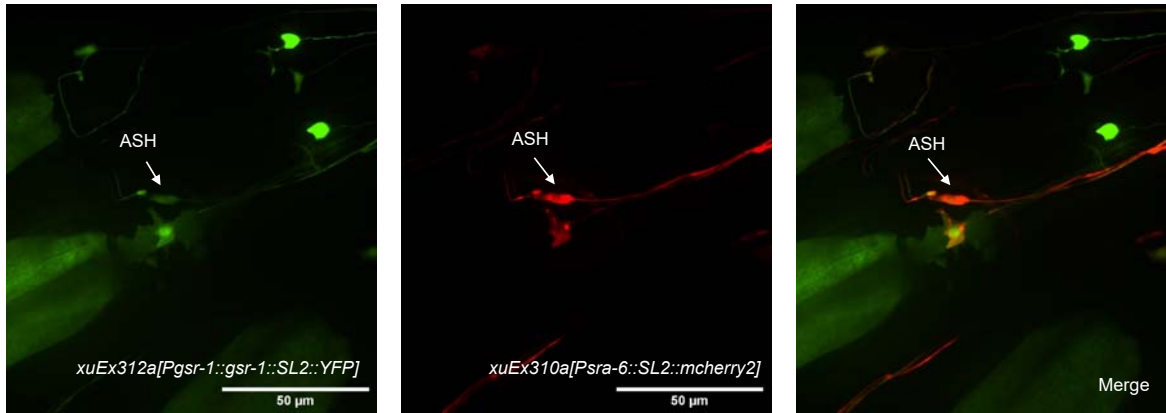

Supplement: S5 Fig — (A) trxr-1 is expressed in ASH neurons. A genomic fragment (757 bp promoter region) of trxr-1 gene was used to drive the expression of YFP. Psra-6::SL2::mCherry was used to mark ASH neurons. Shown are confocal images. The arrows point to ASH. (B) gsr-1 is expressed in ASH neurons. A ~2.3 kb genomic fragment of gsr-1 gene (324 bp promoter and the entire coding region) was used to drive the expression of YFP. Psra-6::SL2::mCherry was used to mark ASH neurons. Shown are confocal images. The arrows point to ASH. (PDF) [file pgen.1009257.s005.pdf]
